# 3D printing-based minimally invasive cannulated screw treatment of unstable pelvic fracture

**DOI:** 10.1186/s13018-018-0778-1

**Published:** 2018-04-04

**Authors:** Leyi Cai, Yingying Zhang, Chunhui Chen, Yiting Lou, Xiaoshan Guo, Jianshun Wang

**Affiliations:** 1 Department of Orthopaedics Surgery, The Second Affiliated Hospital and Yuying Children’s Hospital of Wenzhou Medical University, NO.109, XueYuan West Road, Luheng District, Wenzhou, 325000 Zhejiang Province People’s Republic of China; 2 Department of Radiology, The Second Affiliated Hospital and Yuying Children’s Hospital of Wenzhou Medical University, NO.109, XueYuan West Road , Luheng District, Wenzhou, 325000 Zhejiang Province People’s Republic of China

**Keywords:** Pelvic fracture, Minimally invasive cannulated screw fixation, 3D printing

## Abstract

**Background:**

Open reduction and internal fixation of pelvic fractures could restore the stability of the pelvic ring, but there were several problems. Minimally invasive closed reduction cannulated screw treatment of pelvic fractures has lots advantages. However, how to insert the cannulated screw safely and effectively to achieve a reliable fixation were still hard for orthopedist. Our aim was to explore the significance of 3D printing technology as a new method for minimally invasive cannulated screw treatment of unstable pelvic fracture.

**Methods:**

One hundred thirty-seven patients with unstable pelvic fractures from 2014 to 2016 were retrospectively analyzed. Based on the usage of 3D printing technology for preoperative simulation surgery, they were assigned to 3D printing group (*n* = 65) and control group (*n* = 72), respectively. These two groups were assessed in terms of operative time, intraoperative fluoroscopy, postoperative reduction effect, fracture healing time, and follow-up function. The effect of 3D printing technology was evaluated through minimally invasive cannulated screw treatment.

**Results:**

There was no significant difference in these two groups with respect to general conditions, such as age, gender, fracture type, time from injury to operation, injury cause, and combined injury. Length of surgery and average number of fluoroscopies were statistically different for 3D printing group and the control group (*p* < 0.01), i.e., 58.6 vs. 72.3 min and 29.3 vs. 37 min, respectively. Using the Matta radiological scoring systems, the reduction was scored excellent in 21/65 cases (32.3%) and good in 30/65 cases (46.2%) for the 3D printing group, versus 22/72 cases (30.6%) scored as excellent and 36/72 cases (50%) as good for the control group. On the other hand, using the Majeed functional scoring criteria, there were 27/65 (41.5%) excellent and 26/65 (40%) good cases for the 3D printing group in comparison to 30/72 (41.7%) and 28/72 (38.9%) cases for the control group, respectively. This suggests no significant difference between these two groups about the function outcomes.

**Conclusion:**

Full reduction and proper fixation of the pelvic ring and reconstruction of anatomical morphology are of great significance to patients’ early functional exercise and for the reduction of long-term complications. This retrospective study has demonstrated the 3D printing technology as a potential approach for improving the diagnosis and treatment of pelvic fractures.

**Trial registration:**

The study was retrospectively registered at the Chinese Clinical Trial Registry, number: ChiCTR-TRC-17012798, trial registration date: 26 Sept. 2017.

## Background

The goal in treating pelvic fractures is to reduce the pelvis, restore the stability of the pelvic ring, and reduce the incidence of complications [1, 2]. Open reduction and internal fixation of pelvic fractures are associated with several problems, such as great damage, increased bleeding, and nerve and vascular injury, as well as a high incidence of complications, such as wound disunion and infection. In contrast, the minimally invasive closed reduction cannulated screw treatment of pelvic fractures has advantages such as small incision, reliable fixation, and low cost. In addition, it can effectively shorten the length of surgery and reduce damage to the nerves and blood vessels in the surgical region [3–5].

Currently, several difficulties in the treatment of pelvic fractures are still remaining. For example, how to fully restore the anatomical structure of the fractured pelvis through closed reduction and how to insert the cannulated screw safely and effectively to achieve a reliable fixation, thereby reducing the damage to the blood vessels and nerves, as well as pelvic organs, owing to screw malposition.

In recent years, along with the development of digital medicine in clinical treatment, 3D printing technology has achieved a quantum leap from virtual simulation to the real-world clinical application [6, 7]. At the same time, the technology has been utilized as an important component of personalized treatment plans in fracture treatment, especially the treatment of unstable pelvic fractures, which has complex requirements with respect to the diagnosis of the overall fracture pattern and surgical procedures [8]. Most importantly, the positioning of the cannulated screw in the process of minimally invasive treatment of unstable pelvic fractures is very critical. Therefore, accurate diagnosis and perfect preoperative planning are essential to the whole treatment. The emergence of 3D printing technology undoubtedly aids in solving this orthopedic problem.

In this retrospective study, data on patients treated for unstable pelvic fracture were collected from the Trauma Orthopedics Department of the Second Affiliated Hospital of Wenzhou Medical University. All patients were treated in the department from January 2014 to January 2016. We adopted a 3D printed physical model for preoperative planning and physical simulation of diagnosis and treatment of unstable pelvic fractures. Its feasibility and significance were discussed and compared with conventional surgical methods.

## Methods

An observational, retrospective study design was implemented using the data obtained from patient records and radiological and functional recovery follow-up examinations.

The study was approved by the Ethical Review Boards of the The Second Affiliated Hospital and Yuying Children’s Hospital of Wenzhou Medical University to evaluate the short-term results of 3D printing-based minimally invasive cannulated screw treatment of the unstable pelvic fracture. (No. L-2014-0*, Date 28/7/2014). The written consent to participate was obtained from the participants.

### Inclusion and exclusion criteria

The inclusion criteria were (1) type B or type C pelvic fracture according to Tile classification and received minimally invasive cannulated screw fixation surgery, (2) 18 years or older, (3) closed fractures, (4) injury time < 2 weeks, and (5) participated in all follow-up sessions and had complete radiographic data.

The exclusion criteria were (1) open fracture; (2) multiple fractures, or unable to undergo surgical treatment within 2 weeks owing to combined damage of the blood vessels, nerves, brain, abdominal cavity, and pleural cavity; (3) severe primary disease of the heart, brain, liver, kidney, lung, or hematopoietic system; and (4) lost to follow-up or incomplete radiographic data.

### Patients

A total of 137 patients were included in this study, of whom 65 patients (37 men and 28 women) were treated with the assistance of a 3D printed 1:1 scale physical model for preoperative planning and simulation of cannulated screw implantation (3D printing group). The remaining 72 patients (45 men and 27 women) were treated with conventional surgery without using the 3D printing technique (control group). Pelvic fractures were first analyzed using Tile classification [9]. In the 3D printing group, 43 cases were type B fracture and 22 cases were type C fracture. In the control group, the numbers of type B and type C fractures were 47 and 25, respectively. Table 1 shows the general data of patients in these two groups; no significant difference was identified between these two groups, meaning they are comparable.

**Table 1:** General data of patients

Group	No. of patients	Age (years)	Sex	Fracture Tile classification	Time from injury to operation (days)	Injury cause	Combined injury
3D printing group	65	33.08 ± 4.91	Male: 37 Female: 28	B: 43 C: 22	8.49 ± 3.07	Traffic: 38 Falling: 22 Blunt force: 5	Brain: 9 Chest: 12 Abdomen: 15 None: 29
Control group	72	32.63 ± 4.72	Male: 45 Female: 27	B: 47 C: 25	8.71 ± 2.97	Traffic 43 Falling: 25 Blunt force: 4	Brain: 11 Chest: 15 Abdomen: 17 None: 29
Test value		*t* = 0.549	*Χ*^2^ = 0.442	*Χ*^2^ = 0.012	*t* = 0.418	*Χ*^2^ = 0.254	*Χ*^2^ = 0.301
*p*		0.584	0.506	0.914	0.676	0.881	0.96

### Preoperative diagnosis

Traumatic control was carried out on admission. The pelvic volume was usually constrained by the pelvic girdle; this was accompanied with the treatment of complications and advanced trauma life support [10]. Pelvic X-ray plain film and thin-layer computed tomography data were routinely obtained after admission.

### 3D printing group

Computed tomography was prescribed, and DICOM computed tomography data were imported into Mimics18.0 (Materialise, Belgium) for 3D reconstruction. A 1:1 scale 3D model was printed using a 3D printer (“Rapid solid 3D forming system based on orthopedic medical image reconstruction” provided by Changzhou Watson 3D Printing Research Institute Co., Ltd.) in the Institute of Orthopedic Digitization in the hospital.

Prior to surgery, the 1:1 scale model and radiographic data were used as a reference for surgeons to assess the anterior and posterior fracture parts and the degree of displacement.

**Fig. 1:**
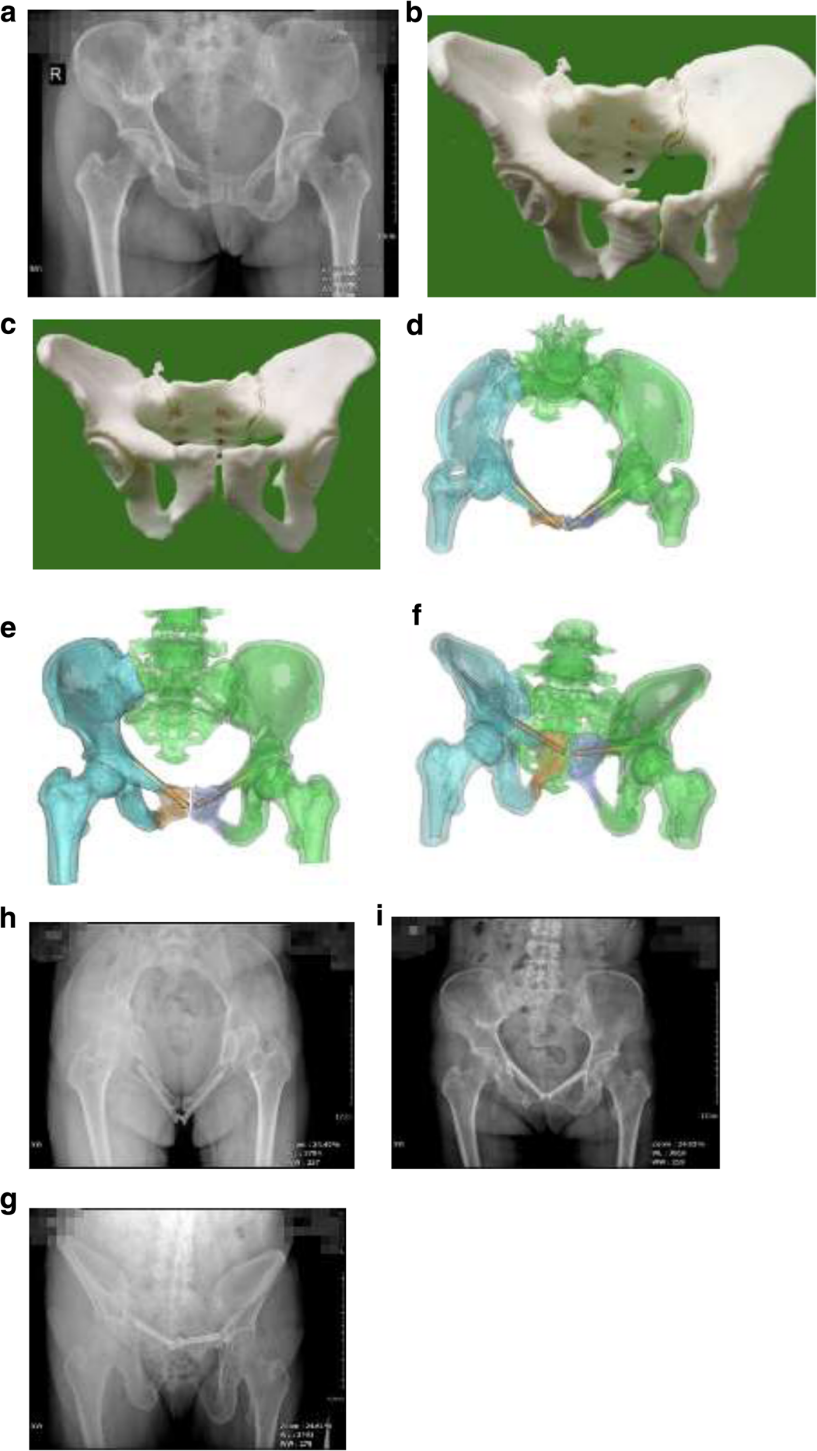
Tile type B pelvic fracture in the 3D printing group, bilateral upper and lower pubic fracture, sacral compression fractures. **a** preoperative X-ray. **b** 3D printed 1:1 scale physical model. **c** 3D printed 1:1 scale physical model after simulated reduction. **d–f** Digitally designed superior pubic retrograde cannulated screw. **h**–**g** X-ray after reduction and screw implantation referring to the 3D physical model. The outcome of reduction was excellent

For the surgical simulation and preoperation, each fracture site of the 3D printed model was digitally separated. Using the contralateral healthy hemi-pelvis as a reference, the reduction procedure was simulated with Kirschner wire. The Kirschner wire was used to simulate the implantation of the cannulated screw. Its position and direction were then determined according to the surgical position. Closed reduction sacroiliac screw fixation was used for posterior pelvic ring fractures that required surgical fixation. Trans-pubic symphysis screws or pubic retrograde cannulated screws were used for anterior pelvic ring fractures. Hence, preoperative routine preparation was improved (Fig 1).

### Control group

The treatment plan for patients in the control group was basically the same as that for the 3D printing group. After the vital signs had improved in the emergency department, the degree of fracture and displacement was determined based on conventional X-ray film and 2D and 3D computed tomography data; thereafter, the surgical plan was made by the same surgeons. The surgical procedures and fixation method were the same as the 3D printing group.

### Surgical methods

General anesthesia was administered. The surgical position was decided individually according to the fracture displacement.

#### Fixation of posterior pelvic ring fracture

For patients with sacroiliac joint separation or severe sacral fracture, lower limb traction and temporary placement of a Schanz pin in the bilateral iliac crest were carried out to control the reduction under anesthesia. When a satisfied reduction was achieved, a Kirschner pin was used to fix the pelvis temporarily. The sacroiliac screw was placed with the patient in the prone position. For the 3D printing group, a conventional sacroiliac screw was implanted, referring to the position and direction of the preset cannulated screw during the preoperative planning on the 3D model. For the control group, the screw was implanted using traditional surgical methods.

#### Treatment of anterior pelvic ring injury

**Fig. 2:**
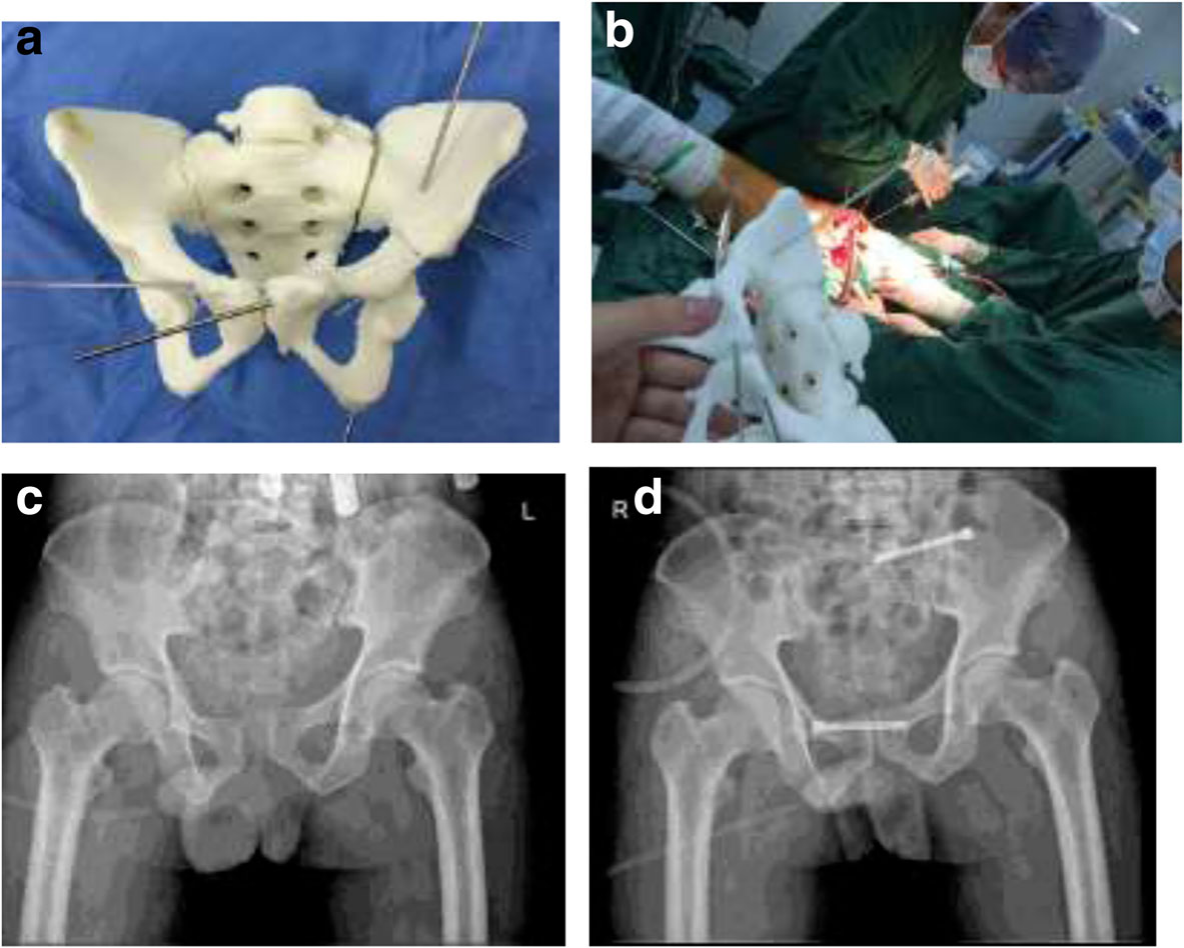
**a** 3D printed 1:1 scale physical model after simulated reduction; the Kirschner wire shows the simulated position and direction of the screw. **b** 3D printed 1:1 scale physical model used as a reference in operation. **c** Preoperative X-ray showing type B pelvic fracture, separation of the pubic symphysis, and dislocation of the left sacroiliac joint. **d** Postoperative pelvis showing pubic symphysis fixation with horizontal cannulated screw and left sacroiliac joint cannulated screw fixation

All patients were treated in the supine position. A small incision of the pubic symphysis and cannulated retrograde screw implantation were performed for patients with pubic ramus fracture. For patients with separation of the pubic symphysis, the pubic symphysis was fixed with a percutaneous trans-pubic screw through a small incision. For the 3D printing group, the screw was implanted referring to the position and direction of the cannulated screw determined in the preoperative planning with the 3D model. For the control group, screw implantation was performed according to traditional surgical methods (Fig 2).

### Evaluation criteria

Length of surgery, number of fluoroscopies, postoperative reduction effect, perioperative complications, time of fracture healing, and functional score were recorded and analyzed for all patients. The reduction of pelvic fractures was evaluated using the Matta radiological scoring system [11]. The functional status at the last follow-up was assessed using the Majeed functional scoring criteria [12]. SPSS 15.0 statistical software (SPSS, USA) was used for statistical analysis; significance was assumed for *p* < 0.05.

## Results

There was no significant difference in the two groups with respect to general condition, such as age, gender, fracture type, time from injury to operation, injury cause, and combined injury, so they were comparable (Table 1, *p* > 0.05). The patients in the two groups were followed up for an average period of 9 months (range 6 to 15 months).

In the 3D printing group, there were 37 males and 28 females, with a mean age of 33.08. Among those cases, there were 43 type B fractures and 22 type C fractures according to the Tile classification. Traffic accident (38 fractures) was the primary injury mechanism, followed by falling accident (22 fractures), and others (5 fractures). In the control group, it consisted of 45 males and 27 females, with a mean age of 32.63. Forty-seven type B fractures and 25 type C fractures were included. Similarly, the injury mechanism falls into traffic accident (43 fractures), falling (25 fractures), and others (4 fractures). Surgical treatment was performed 5 to 12 days after injury (average 8.5 days) in the 3D printing group and 4 to 12 days (average 8.7 days) after injury in the control group.

The average surgery length was 58.63 ± 13.38 min and 72.38 ± 13.4 min in the 3D printing group and the control group, respectively. The average number of fluoroscopies was 29.31 ± 3.56 in the 3D printing group and 36.63 ± 2.83 in the control group. Both of these two parameters are statistically significant (*p* < 0.001). The quality of pelvic reduction was assessed according to the Matta scoring criteria. In the 3D printing group, excellence was demonstrated in 21/65 (32.3%) cases and good in 30/65 (46.2%) cases, together accounting for 78.5% of patients in the group. In the control group, there were 22/72 (30.6%) excellent and 36/72 (50%) good cases, together accounting for 80.6% of the group. During the last follow-up session, efficacy was evaluated according to the Majeed function score. For the 3D printing group, excellence was demonstrated in 27/65 (41.5%) cases and 26/65 (40%) cases scored good, resulting in an excellent-or-good rate of 81.5%. For the control group, the number of excellent and good cases was 30/72 (41.7%) and 28/72 (38.9%), respectively. These two together accounted for 84.7% of total patients in the group. There was no significant difference between the two groups regarding the function outcomes. No patient had fracture nonunion or iatrogenic vascular/nerve injury after the surgery, as shown in Table 2.

**Table 2:** Comparison of surgical data

Group	Duration of surgery (min)	Time of fluoroscopies	Fracture healing time (weeks)	Majeed excellent-or-good score rate	Matta excellent-or-good score rate
3D printing group	58.63 ± 13.38	29.31 ± 3.56	14.5 ± 1.56	81.5% (53/65)	78.5% (51/65)
Control group	72.38 ± 13.4	36.63 ± 2.83	13.8 ± 1.96	84.7% (58/72)	80.6% (58/72)
Test value	*t* = 5.99	*t* = 13.37	*Χ*^2^ 0.438	*Χ*^2^ = 0.021	*Χ*^2^ = 0.092
*p*	0.00*	0.00*	0.766	0.884	0.762

* means that there is statistical difference between the two groups

## Discussion

Pelvic fracture is often secondary to high-energy trauma, predominantly caused by traffic accidents and high fall injuries, and is often accompanied with other injuries, thus leading to high mortality and morbidity [13]. According to the traditional classification, Tile type B rotational and type C vertical pelvic fractures are unstable pelvic fractures. Emergent trauma control and restoration of pelvic ring stability have a positive effect in saving patients’ lives [14]. For most unstable pelvic fractures which feature complex spatial displacements, a surgical treatment of reduction and fixation is often necessary to prevent long-term dysfunction due to malunion. As the recognized surgical method, closed reduction and minimally invasive cannulated screw fixation are favored by a large number of clinical orthopedic surgeons at present, yet this procedure has complex requirements for fracture diagnosis and surgical operation [15].

A large number of previous studies have proposed that closed reduction and minimally invasive cannulated screw fixation are effective treatments of unstable pelvic fractures; however, the major risk lies in the injury to the blood vessels, nerves, and pelvic organs, as well as failure of fixation, owing to inaccurate position and length of the cannulated screw. Therefore, how to better determine the needle channel, and place the cannulated screw safely, accurately, and effectively, is the challenge of minimally invasive treatment of pelvic fractures. Although a real-time navigation system could provide certain guidance for implantation of the cannulated screw, it depends on high-performance hardware, which restricts its application [16].

As an important part of current medical development, digital medicine is playing a key role in the clinical diagnosis and treatment of orthopedic problems. 3D printing technology has been applied in orthopedic clinics because of the advantages such as patient-specific physical simulation, accuracy, and validity.

Previous studies suggested that 3D printing of a fracture model is of positive significance in the clinical application of orthopedics, as it could help improve preoperative preparation and the development of a personalized surgical plan and reduce difficulties for clinicians by shortening the learning curve [17]. The emergence and application of a 3D printed 1:1 scale physical model provides a new method of improving the pelvic treatment. Orthopedic surgeons could also preoperate on the key parts of the screw plate position using the 3D model, achieving simulation of pre-surgical planning. During the surgery, screw implantation was simulated and the bone block was determined according to the patient’s position and the direction of internal fixation instruments, which could greatly improve the accuracy and safety of screw implantation.

Postoperative radiographic and functional scores were assessed for the patients in the 3D printing group, demonstrating an excellent-or-good rate of about 80%, which is close to the advanced level worldwide [18]. The surgery length and number of fluoroscopies in the 3D printing group are shorter to those in the control group, indicating that 3D printing technology could serve as a promising new treatment tool during preoperative design and intraoperative reference of pelvic fracture surgery.

The application of 3D printing technology provides accurate and detailed preoperative diagnosis and treatment programs as well as in-depth understanding of displacement and extent of fractures, which is beneficial in proposing a complete diagnostic and treatment plan. As a 1:1 scale physical reference, the 3D model could facilitate a mimicked screw implantation and reduce surgery time and the number of fluoroscopies, which enables a minimally invasive treatment and effectively improves the surgical safety.

## Conclusion

Full reduction and proper fixation of the pelvic ring together with the reconstruction of anatomical morphology are of great significance to patients’ early functional exercise and the reduction of long-term complications. This retrospective study has demonstrated the 3D printing technology as a potential approach for improving the diagnosis and treatment of pelvic fractures.

## Abbreviations

3D: Three-dimensional
DICM: Digital image correlation method
